# Recent advances on catalysts for photocatalytic selective hydrogenation of nitrobenzene to aniline

**DOI:** 10.3389/fchem.2023.1162183

**Published:** 2023-03-10

**Authors:** Jiawen Guo, Huimin Liu, Yuqiao Li, Dezheng Li, Dehua He

**Affiliations:** ^1^ School of Chemical and Environmental Engineering, Liaoning University of Technology, Jinzhou, China; ^2^ Innovative Catalysis Program, Key Lab of Organic Optoelectronics and Molecular Engineering of Ministry of Education, Department of Chemistry, Tsinghua University, Beijing, China

**Keywords:** photocatalysis, selective hydrogenation of nitrobenzene, semiconductor, plasmonic metal-based catalyst, dye

## Abstract

Selective hydrogenation of nitrobenzene (SHN) is an important approach to synthesize aniline, an essential intermediate with extremely high research significance and value in the fields of textiles, pharmaceuticals and dyes. SHN reaction requires high temperature and high hydrogen pressure *via* the conventional thermal-driven catalytic process. On the contrary, photocatalysis provides an avenue to achieve high nitrobenzene conversion and high selectivity towards aniline at room temperature and low hydrogen pressure, which is in line with the sustainable development strategies. Designing efficient photocatalysts is a crucial step in SHN. Up to now, several photocatalysts have been explored for photocatalytic SHN, such as TiO_2_, CdS, Cu/graphene and Eosin Y. In this review, we divide the photocatalysts into three categories based on the characteristics of the light harvesting units, including semiconductors, plasmonic metal-based catalysts and dyes. The recent progress of the three categories of photocatalysts is summarized, the challenges and opportunities are pointed out and the future development prospects are described. It aims to give a clear picture to the catalysis community and stimulate more efforts in this research area.

## 1 Introduction

Aniline is one of the important amine substances and an essential intermediate for the synthesis of organic chemical raw materials. It is mainly used for the production of textiles, medicine, rubber auxiliaries, dyes and others, and has extremely high research significance and value (Junhua et al., 2010; Maji et al., 2011; Jiang et al., 2015; Wang et al., 2015; Kou et al., 2017). Aniline could be synthesized *via* the following approaches, such as chlorophenyl amination (Chen et al., 2009), direct ammonization (Wang et al., 2019a) and selective hydrogenation of nitrobenzene (SHN) (Wei et al., 2014). Notably, the selectivity towards aniline in SHN is high since few side reactions accompany the SHN main reaction, which makes it as the most simple, clean and efficient approach for the synthesis of aniline.

In a conventional thermal-driven catalytic process, SHN requires high temperature and high hydrogen pressure to achieve high yields of aniline. On the contrary, photocatalysis can realize high nitrobenzene conversion and high selectivity to aniline at room temperature and low hydrogen pressure (Tsutsumi et al., 2016; Jiang et al., 2021), which reduces energy consumption and is in line with the sustainable development strategies.

**SCHEME 1:**
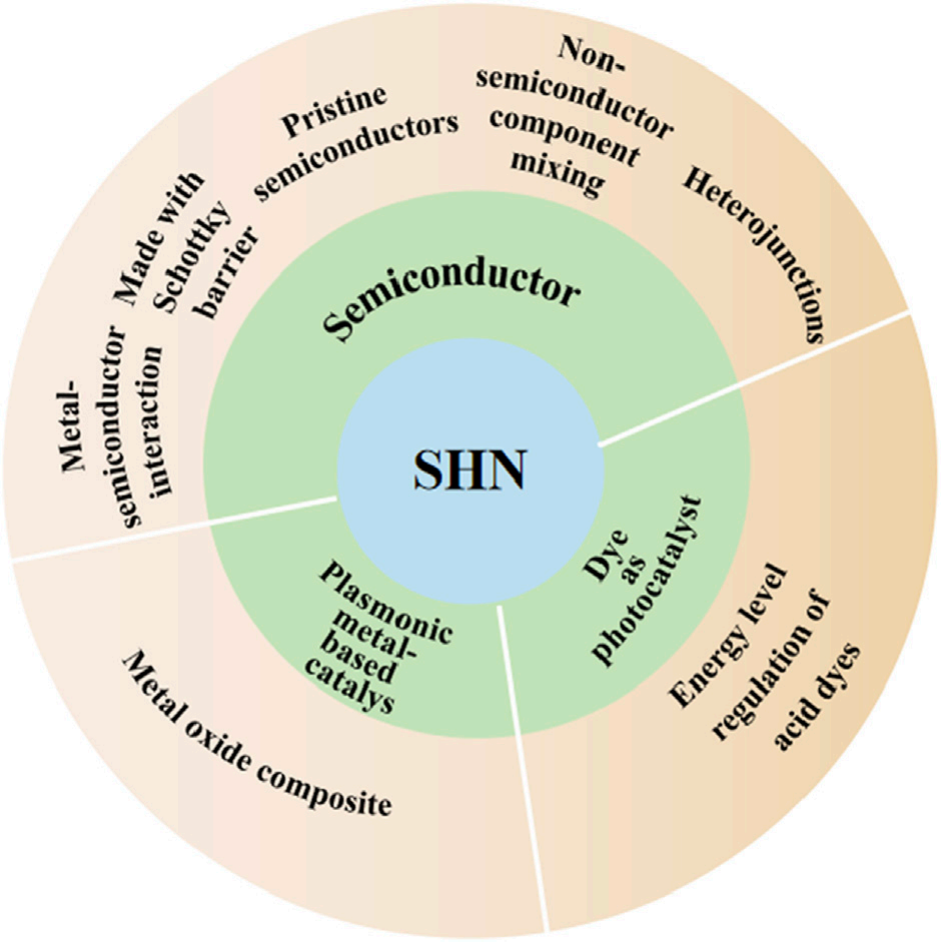
Strategies for photocatalytic selective hydrogenation of nitrobenzene to aniline.

In most chemical reactions, catalysts play important roles in reducing the activation energies and increasing the reaction rates. Researchers are committed to developing an efficient SHN photocatalyst. Up to now, several photocatalysts for photocatalytic SHN reaction have been developed, including TiO_2_, CdS, Cu/graphene, and Eosin Y. In this review, we divide the photocatalysts into three categories based on the nature of the light harvesting units, which are semiconductors, plasmonic metal-based catalysts and dyes (Scheme 1), respectively. The progress of photocatalysts for SHN are reviewed and mechanisms of various catalysts are illustrated, aiming at providing a methodical summary and inspiring more researches to develop the fields of photocatalysis and nitrobenzene hydrogenation reaction.

## 2 Semiconductor

Since the discovery of photocatalysis by Fujishima et al. in 1972, the field of semiconductor photocatalysis has been opened up, the applications of semiconductors in a wide variety of reactions have been extensively studied, and the mechanism of semiconductors in photocatalytic reactions have been generalized (Akhundi et al., 2022; Murali et al., 2022; Fujishima et al., 2000).

The energy band structure of a semiconductor is usually composed of a low-energy valence band (VB) filled with electrons and an empty high-energy conduction band (CB). When light is irradiated onto a semiconductor, if the energy of the photon is greater than or equal to the semiconductor band gap (BG), the electrons in VB of the semiconductor will be excited to CB, and meanwhile, holes are generated in VB (Fujishima et al., 2000; Xuan and Xiao, 2012; Wang et al., 2019b).

Electrons and holes generated by photoexcitation migrate to the surface of the semiconductor for redox reactions. The photogenerated holes in VB have a strong oxidizing ability, which can capture the adsorbed substances on the surface of the semiconductor or electrons in the solvent (served as scavengers) and oxidize low-valent ions into high-valent ions. The electrons transitioning to CB are of strong reducing ability, which can be received by the electron acceptor on the semiconductor surface or holes in the solvent (served as scavengers) and reduce high-valent ions to low-valent ions (Fukui et al., 2019; Roy, 2020; Yun et al., 2020). For SHN, the electrons undergo the reduction half reaction with nitrobenzene adsorbed on the surface while holes oxidize the adsorbed hydrogen species or hole scavengers.

**SCHEME 2:**
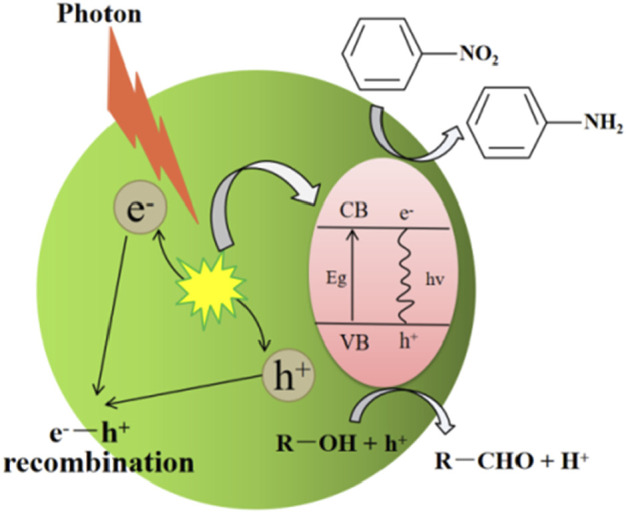
Mechanism of SHN catalyzed by a semiconductor, where alcohol (R-OH) is used as a hole scavenger.

In terms of thermodynamics, the photocatalytic reaction requires that the redox potential of the electron donor is lower than the redox potential of the VB, while the redox potential of the electron acceptor should be higher than the redox potential of the CB (Buzzetti et al., 2019). Theoretically, when the CB position of a semiconductor is lower than −0.486 V, nitrobenzene can be photocatalytically reduced to aniline (E^θ^(C_6_H_5_NO_2_/C_6_H_5_NH_2_) = −0.486 V, vs. NHE). The oxidation reaction potential on electron acceptor depends on the type of reaction environment. If hydrogen is used as a reactant and directly oxidized at VB, the VB position of a semiconductor should be higher than 0 V (E^θ^(H^+^/H) = −0 V, vs. NHE) (Gao et al., 2020). In the case that alcohols or sodium sulfite are used as scavengers, the VB position of a semiconductor should be higher than their redox potentials. For example, when methanol is used as a hole scavenger and it is oxidized to formaldehyde (E^θ^(CH_3_OH/CH_2_O) = +2.62 V, vs. NHE), VB position should be higher than +2.62 V (Shen et al., 2021). The reaction mechanism of SHN is shown in Scheme 2, where alcohol is used as a hole scavenger.

Noteworthy, only the electrons and holes participated in redox reactions are beneficial for the photocatalytic performance. Some electrons and holes generated by photoexcitation may also recombine inside or on the surface of the semiconductor within a few milliseconds. Therefore, to improve the ability of the catalyst in the photoreduction of nitrobenzene, it is important to make full utilization of the photoexcited electron hole pairs and minimize the recombination of electrons and holes (Aljahdali et al., 2017; Abdullah et al., 2020).

Up to now, a series of semiconductors have been applied in photocatalytic SHN. In this section, we will describe the progress of photocatalytic SHN in each type of semiconductor photocatalysts as well as the modification approaches to improve their performance.

### 2.1 TiO_2_

TiO_2_ has been extensively studied in the field of photocatalysis due to its following advantages (Daghrir et al., 2013; Zhou et al., 2022): ➀ It is low-cost, safe and non-toxic; ➁ It has good light and chemical stability, and no photocorrosion or chemical corrosion will occur during the photocatalytic reactions; ➂ It has a suitable BG, which allows it to be excited by ultraviolet light; ➃ The photogenerated charges are of strong redox ability, which endows TiO_2_ relatively high photocatalytic activity under ultraviolet irradiation (Tamaki et al., 2007; Qiu et al., 2014; Chaturvedi and Singh, 2021). These advantages of TiO_2_ make it promising for photocatalytic SHN (Kandiel et al., 2014; Oliveira et al., 2016; Wang et al., 2016).

**FIGURE 1:**
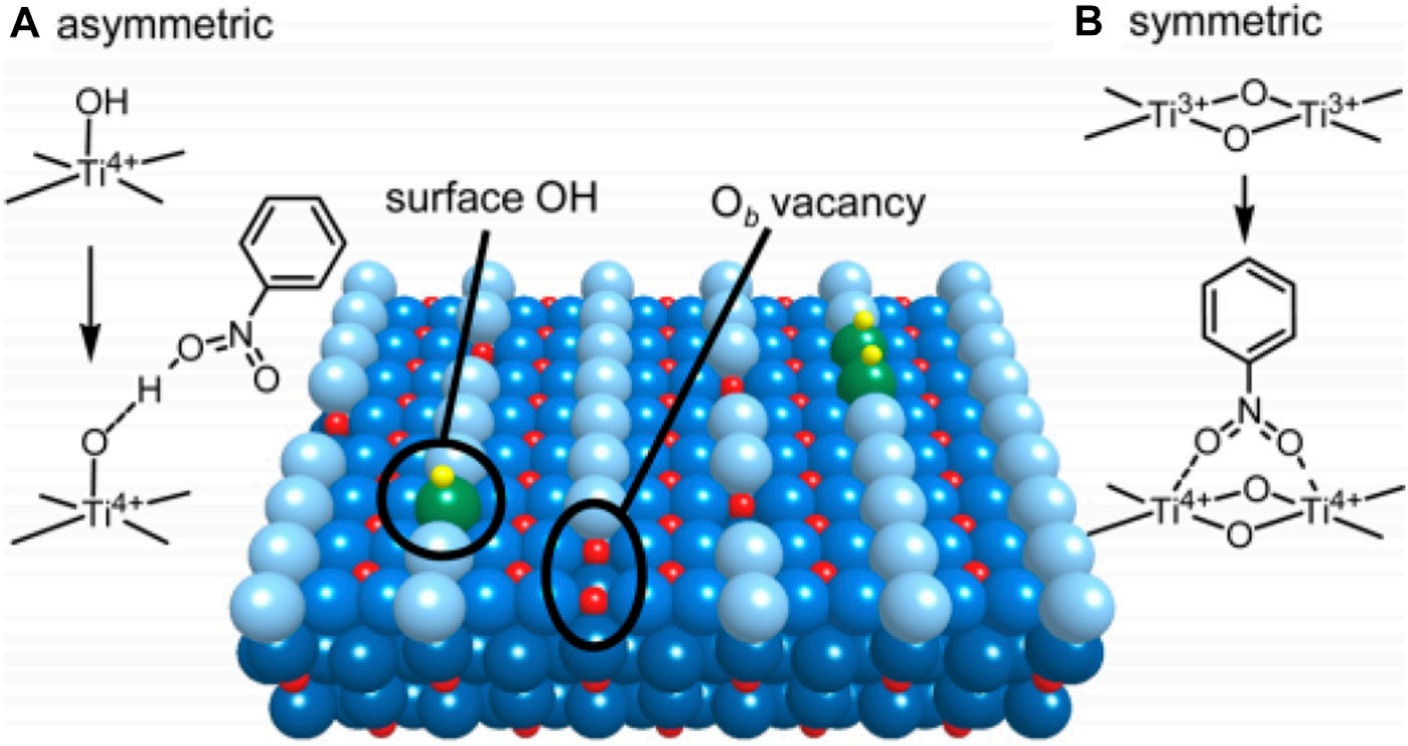
Surface structure of rutile TiO_2_ (Yang et al., 2022). **(A)** Asymmetric adsorption mode of nitrobenzene, and **(B)** symmetric adsorption mode of nitrobenzene. Green and light blue spheres: O_b_ atom at [001] azimuth; yellow and red spheres: H and Ti atoms. Reproduced with permission from reference (Shiraishi et al., 2012).

Shiraishi et al. (2012) research on the application of pristine TiO_2_ in photocatalytic SHN is a typical example. The authors investigated the influence of crystalline structure of TiO_2_ on the catalytic performance in SHN, where ethanol was used as the hydrogen source (Shiraishi et al., 2012). It suggested that commercial P25 and anatase TiO_2_ were poor in SHN, resulting in unsatisfactory performance. On the contrary, rutile TiO_2_ exhibited relatively high catalytic performance, with an almost complete nitrobenzene conversion and a selectivity towards aniline higher than 90.0%. Characterization results revealed that Ti atoms in the surface defects of rutile TiO_2_ were the active sites. Rutile TiO_2_ (Yang et al., 2022) surface was an alternating arrangement of coordinated 5-fold Ti^4+^ atoms and bridging O^2−^ atoms (O_b_) running along the (001) direction (Figure 1) (Shiraishi et al., 2012). O_b_ vacancies were the surface defects, in which two excessive electrons related to O_b_ were transferred to the empty three-dimensional orbitals of adjacent Ti^4+^ atoms, resulting in two Ti^3+^ atoms. On the one hand, the adsorption sites of nitrobenzene were formed by surface Ti^3+^ atoms through electron transfer and served as capture sites for the light-formed CB e−. On the other hand, the Ti^3+^ atom on the surface of TiO_2_ acted as the adsorption site of nitroaromatic hydrocarbons, and was the capture center of electron e− in the photoconduction band. Ti^3+^ atoms on the surface were both adsorption sites and electron capture centers. These two effects rapidly hydrogenated the nitrobenzene on the surface Ti^3+^ atoms to aniline and accelerated the reaction (Shiraishi et al., 2012).

Although rutile TiO_2_ could catalyze photocatalytic SHN, it could only absorb ultraviolet light and the light utilization efficiency is low, which could not meet expectations. Therefore, further modification is necessary. At present, plenty of modification approaches have been developed and applied to the modification of pristine semiconductors, including fabricating a catalyst with Schottky barrier, constructing a heterojunction photocatalyst with another semiconductor and constructing a hybrid catalyst with non-semiconductor component.

➀ Fabricating a catalyst with Schottky barrier. Metals and semiconductors can form a Mott-Schottky junction, which is a simple metal-semiconductor interface, where electrons flow directionally between the metal and the semiconductor, with non-linear impedance characteristics (Lin et al., 2019; Kw et al., 2020; Žerjav et al., 2021). Schottky proposed a metal-semiconductor contact barrier model under ideal conditions that ignores the interface state, so it is called the Schottky barrier (Xu et al., 2023). Catalysts with Schottky barrier are generally of strong capacity to improve the separation of electron hole pairs, thereby exhibiting superior catalytic performance. In addition, the deposition of metals (for instance Au, Ag, Pt, Pd, and Ru) could change the surface properties of semiconductors as well as the deposited metals, enlarge the light absorption range of the catalyst to the visible light region, and ultimately contribute to the catalytic performance. With the maturity of the Schottky contact theory, the research of catalysts based on the Schottky barrier principle has gradually increased. Some typical examples are illustrated below.

**FIGURE 2:**
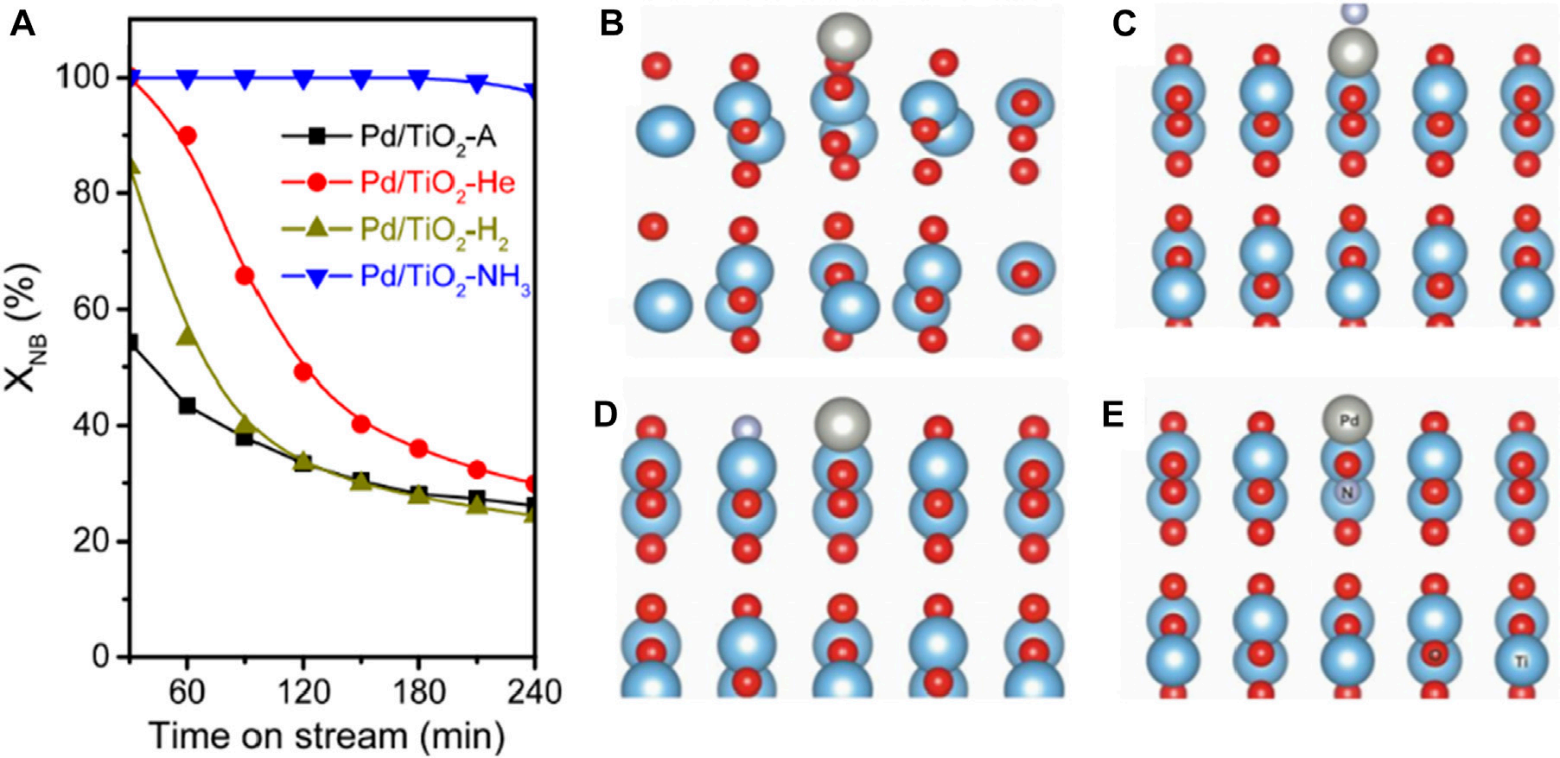
**(A)** Transitory conversion of nitrobenzene over Pd/TiO_2_ catalyst at 160°C. A side view of the optimized structures for Pd/TiO_2_ systems, with a Pd atom deposited on **(B)** non-doped TiO_2_, **(C)** TiO_2_ with N-_adsorption_ on the surface, **(D)** TiO_2_ with N-s_ubstitution_ on the surface and **(E)** TiO_2_ with N-_substitution_ in the subsurface region. Spheres in red: O, light blue: Ti, gray: Pd, light purple: N, and light pink: H. Reproduced with permission from reference (Chen et al., 2017).

The interaction between metal and semiconductor could exert functions on catalytic performance. Chen et al. (2017) treated commercial TiO_2_ in He, H_2_, and NH_3_, respectively, and then deposited Pd nanoparticles on the pristine and modified TiO_2_ and obtained four catalysts (denoted as Pd/TiO_2_-A, Pd/TiO_2_-He, Pd/TiO_2_-H_2_ and Pd/TiO_2_-NH_3_, respectively) for photocatalytic SHN. Pd/TiO_2_-NH_3_ exhibited high catalytic activity (Figure 2A, Pd/TiO_2_-NH_3_ exhibited an initial nitrobenzene conversion of ∼100.0%, much higher than the reference catalysts Pd/TiO_2_-A and Pd/TiO_2_-H_2_), and good stability in photocatalytic SHN (Figure 2A, Pd/TiO_2_-NH_3_ remained stable for at least 240 min; on the contrary, Pd/TiO_2_-A gradually deactivated after 30 min while Pd/TiO_2_-He and Pd/TiO_2_-H_2_ rapidly deactivated after 240 min). It revealed that there was a strong interaction between Pd and the support, which tailored the electronic state of Pd/TiO_2_ catalyst and promoted the activity. Density function theory (DFT) calculations suggested that Pd was positively charged while N was negatively charged (Figures 2B–E). When Pd and N atoms were electronically coupled at the surface, a large number of electrons accumulated around the N nucleus, resulting in Pd-N pairs. Additionally, the doping of N into TiO_2_ had a structure-promoting effect, which favored the dispersion of Pd species on TiO_2_, affording more active sites and expediting the reaction (Chen et al., 2017).

**FIGURE 3:**
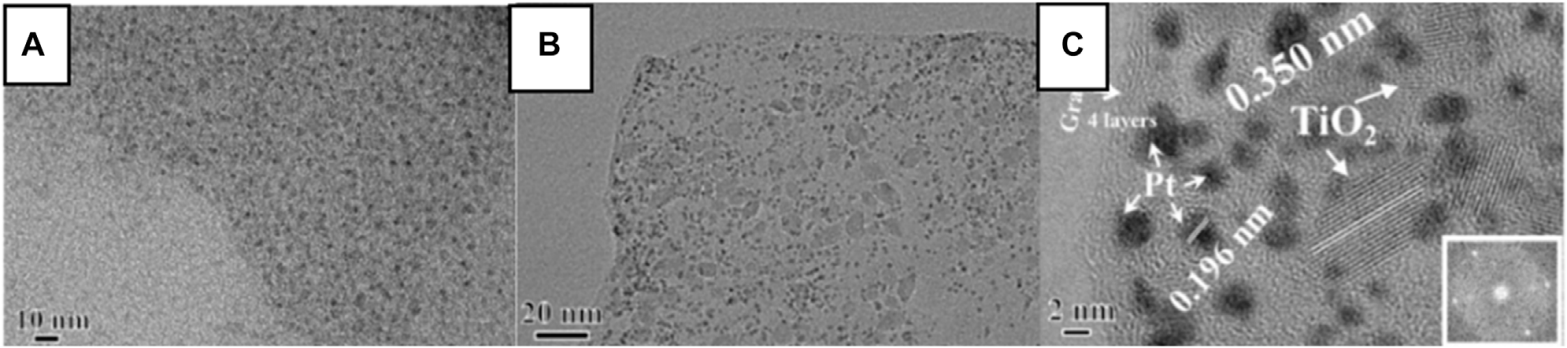
**(A)** High resolution transmission electron microscope (HRTEM) images of TiO_2_-RGO, and **(B)** TEM and **(C)** HRTEM images of the Pt-TiO_2_-RGO catalyst. Reproduced with permission from reference (Qiu et al., 2016).

Schottky barrier could enhance the electron hole separation efficiency and contribute to the catalytic performance (Kamegawa et al., 2012; Qiu et al., 2016). For instance, Kamegawa et al. (2012) modified the surface of Pt-TiO_2_ with colorless 2, 3-dihydroxynaphthalene (2, 3-DN), thereby designing a visible light sensitive photocatalyst for photocatalytic SHN. It is demonstrated that the Pt nanoparticles supported on the surface of TiO_2_ fully separated photogenerated electrons and holes, which endowed the catalyst 2,3-DN/Pt-TiO_2_ high selectivity in photocatalytic SHN. Notably, 2,3-DN(1)/Pt-TiO_2_ (where one describes the content of 2,3-DN) recorded an aniline yield of 75.0%, much higher than other catalysts (such as Pt-TiO_2_ modified by hydroxynaphthalene and 2,3-DN/Pt-TiO_2_ with other 2,3-DN contents). Qiu et al. (2016) designed a ternary-structured Pt-TiO_2_-RGO (RGO: reduced graphene oxide) through sol-gel and microwave-assisted strategies, in which TiO_2_ nanoparticles were grown *in situ* on GO sheets (Figure 3A) and Pt nanoparticles were stabilized on the surface of the TiO_2_-RGO composites (Figure 3B). Over Pt-TiO_2_-RGO catalyst, TiO_2_ existed in the single crystal form (Figure 3C). RGO guaranteed good electrical conductivity of the catalyst, in which RGO not only acted as a stabilizer for TiO_2_ nanocrystals and Pt nanoparticles but also served as a “power wire” that prevented electron and hole recombination. Under sunlight irradiation, the recombination rate of photogenerated electron hole pairs decreased significantly and the photogenerated electrons could be efficiently transferred from TiO_2_ nanocrystals to heterogeneous Pt nanoparticles. As a result, in photocatalytic SHN, under the conditions of 50.0 mg catalyst, 50.0 mL nitrobenzene, 0.6 mL triethanolamine, room temperature and 300 W Xe lamp irradiation, after evaluating for 8.0 h, nitrobenzene conversion and the selectivity towards aniline were 44.0% and 99.0%, respectively. Further extending the reaction time to 20.0 h could boost nitrobenzene conversion and the selectivity towards aniline to 95.0% and 99.0%, respectively.

② Constructing a heterojunction photocatalyst with another semiconductor. A heterojunction is an interface region formed by the contact of two different semiconductors. Heterojunctions often exhibit excellent optoelectronic properties that cannot be achieved by the respective single semiconductors, which poses heterojunction an important position in the field of photocatalysis (Zhou et al., 2018; Ramezanalizadeh and Rafiee, 2020; Yang, 2021). Due to the inconsistency of VB, CB and BG energies, in heterojunctions, the overlap occurs, which promotes the separation of photogenerated electrons and holes, expands the spectral response of TiO_2_, and exhibits better stability and catalytic activity in photocatalytic reactions (Yu et al., 2021; Xu et al., 2022; Zhao et al., 2022).

**FIGURE 4:**
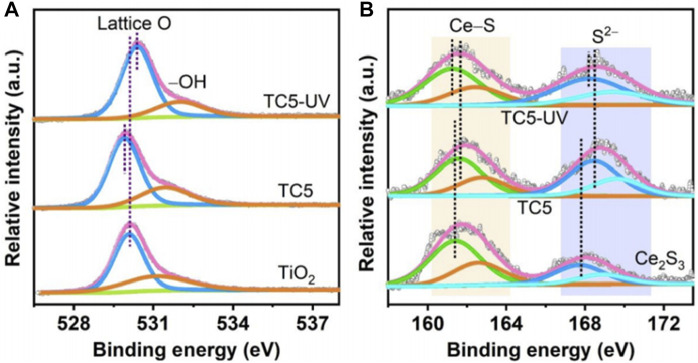
X-ray photoelectron spectroscopy (XPS) spectra of TC_X_ and reference catalysts. **(A)** O 1s of TiO_2_ and TC5, and **(B)** S 2p of Ce_2_S_3_ and TC5. Reproduced with permission from reference (Xu et al., 2021).

The research done by Xu et al. (2021) is a representative work. The authors successfully grew Ce_2_S_3_ nanoparticles on electrospun TiO_2_ nanofibers by a solvothermal strategy (Xu et al., 2021). The resulting Ce_2_S_3_/TiO_2_ heterojunction was named TC_X_ (T: TiO_2_, C: Ce_2_S_3_, x: molar percentage of Ce_2_S_3_ in TC_X_), which served as a photocatalyst for photocatalytic SHN, with water as a proton source. Performance evaluation results indicated that both TiO_2_ nanofibers and Ce_2_S_3_ nanoparticles exhibited low aniline productivity, with aniline yields of 51.0% and 33.0%, respectively; on the contrary, TC_X_ were efficient for aniline production, with the yield of aniline reaching 99.0% when TC5 was used as catalyst under irradiation for 1.5 h. Characterization suggested that in TC5 catalyst, the binding energies of Ti 2p and O 1s were negatively shifted compared with TiO_2_ (Figure 4A) and the binding energies of Ce 3d and S 2p of TC5 were shifted positively relative to that of Ce_2_S_3_ (Figure 4B), indicating that there was electron transfer from Ce_2_S_3_ to TiO_2_ when they were combined without light irradiation. Such an electron transfer between TiO_2_ and Ce_2_S_3_ caused the internal electric field pointing from Ce_2_S_3_ to TiO_2_. In the case that under light irradiation, the binding energies of Ti 2p and O 1s of TC5 were positively shifted, but the binding energies of Ce 3d and S 2p of TC5 were negatively shifted, which confirmed the migration of photoexcited electrons in TiO_2_ CB into Ce_2_S_3_ VB under illumination. Meanwhile, the energy bands of TiO_2_ and Ce_2_S_3_ bent at the interfaces, which drove the efficient separation of photogenerated charge carriers over Ce_2_S_3_/TiO_2_ heterojunction and enhanced its photocatalytic performance.

**FIGURE 5:**
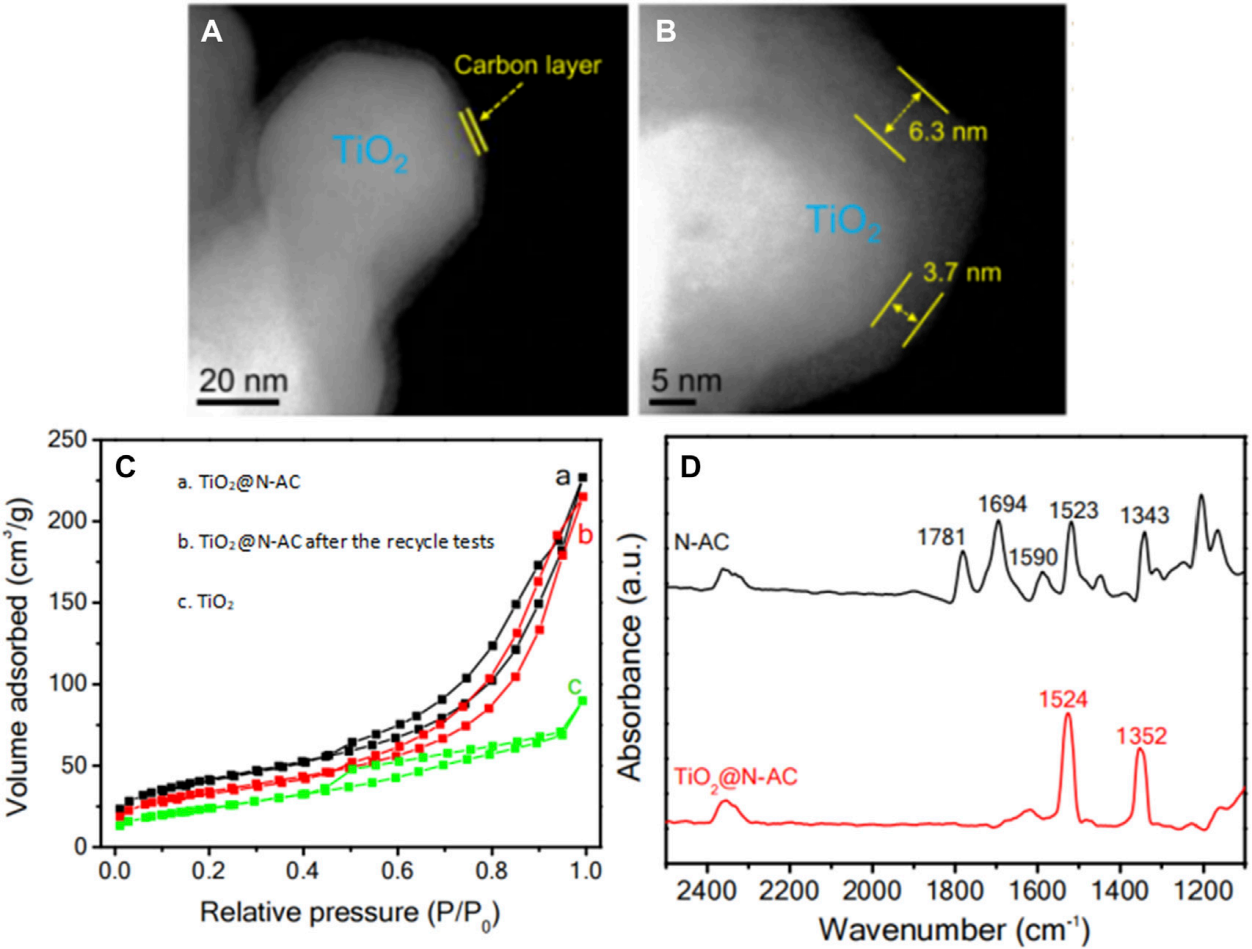
**(A, B)** HAADF-STEM images of TiO_2_@N-AC, **(C)** N_2_ adsorption-desorption isotherms, and **(D)** FTIR spectra of mixed nitrobenzene and benzaldehyde on TiO_2_@N-AC and N-AC. Reproduced with permission from reference (Xu et al., 2019).

➂ Constructing a hybrid catalyst with non-semiconductor component. Hybrid catalysts generally contain active components with different catalytic functions (Prasad et al., 2020; Ahmad et al., 2023). Occasionally, an interface will be formed between the chemical components of the hybrid catalyst, which could improve the overall performance and stability (Han et al., 2018). In this regard, Xu et al. (2019) designed a TiO_2_@N-AC hybrid catalyst, with TiO_2_ as the core and nitrogen-doped carbon (N-AC) as the shell. The thickness of the carbon layers was in the range of 3.5–7.5 nm, evidenced by high-angle annular dark field-scanning transmission electron microscopy (HAADF-STEM) images in Figures 5A, B. Compared with bare TiO_2_ (73 m^2^ g^−1^), TiO_2_@N-AC had a large specific surface area (Figure 5C, 234 m^2^ g^−1^) and abundant mesopores, which facilitated the diffusion of reactant molecules to the surface of TiO_2_ and enhanced the adsorption of nitrobenzene, making TiO_2_@N-AC highly active and selective in the photocatalytic hydrogenation of nitrobenzene. Noteworthy, under the reaction conditions of 0.08 mmol nitrobenzene, 10.0 mg TiO_2_@N-AC catalyst, 4.0 mL isopropanol, reaction temperature 30°C and reaction time 6.0 h, the conversion rate of nitrobenzene reached 99.1% and the selectivity towards aniline reached 98.8%. Moreover, TiO_2_@N-AC also exhibited high conversion and selectivity towards the corresponding functionalized anilines in photocatalytic hydrogenation of nitroarenes with various substituents (such as -Cl, -F, -C=C, C=O, and -C≡N), even better than noble metal-based catalysts (such as Pt, Ag). *In-situ* fourier transform infrared (FTIR) spectrum only gave the band of nitro group when an equivalent amount of nitrobenzene and benzaldehyde were introduced into the TiO_2_@N-AC catalyst (Figure 5D), confirming the unique characteristic of TiO_2_@N-AC to selectively adsorb nitrobenzene, which explained the superior catalytic performance of TiO_2_@N-AC in photocatalytic SHN.

### 2.2 CoS_2_

As one of the representatives of transition metal disulfide, CoS_2_ is considered as a potential photocatalyst due to its low cost, excellent electronic conductivity, thermal stability and photochemical properties (Ahmad et al., 2018; Tang et al., 2019; Zhang et al., 2022), which makes it applicable in photocatalytic SHN. However, the catalytic performance of CoS_2_ is not satisfactory (the conversions of nitrobenzene over CoS_2_ were 72.0% under light irradiation and 23.0% without light irradiation) and needs further improvement (Ma et al., 2017).

**FIGURE 6:**
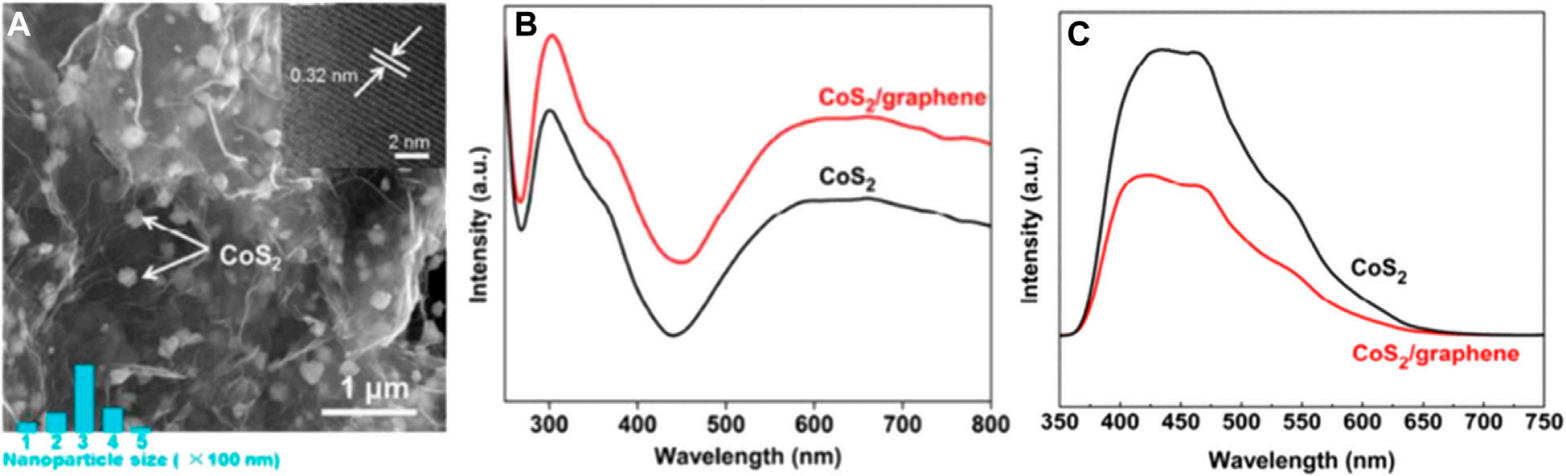
**(A)** Field emission scanning electron microscopy (FESEM) images of CoS_2_/graphene, **(B)** UV-vis diffuse reflectance spectra, and **(C)** photoluminescence spectra of CoS_2_ and CoS_2_/graphene. Reproduced with permission from reference (Ma et al., 2017).

Ma et al. (2017) study is a representative to improve the catalytic performance of CoS_2_ in photocatalytic SHN. The authors prepared a CoS_2_/graphene composite catalyst, by uniformly dispersing CoS_2_ on the graphene sheets (Figure 6A), a material with high electrical conductivity, excellent electron mobility and high surface area. It revealed that, in photocatalytic SHN, over CoS_2_/graphene, under the conditions of 40.0 mg catalyst, 1.0 mmol nitrobenzene, 10.0 mL ethanol, 30°C, 0.25 MPa H_2_ and 300 W Xe lamp irradiation, after evaluating for 1.5 h, nitrobenzene conversion and the selectivity towards aniline reached 99.0% and 100.0%, respectively. Characterization results suggested that CoS_2_/graphene exhibited better light harvesting efficiency, evidenced by the ultraviolet-visible (UV-visible) spectra in Figure 6B, where the absorption at 450–800 nm was attributed to the d-d transitions of Co(II) ions in CoS_2_. Furthermore, when CoS_2_ was supported on graphene, the photogenerated electrons in CoS_2_ could quickly transfer to the conductive graphene sheet, thereby suppressing electron-hole recombination (Figure 6C). Moreover, graphene has a large specific surface area and rich pore structure, which provided more active sites for H_2_ adsorption. According to the above characterization conclusion, it is reasonable to speculate that, on the one hand, H_2_ can be activated by photoexcited holes on the surface of CoS_2_ and form active hydrogen species; On the other hand, nitrobenzene molecules are adsorbed on the catalyst surface, and the N-O bond is also activated by photogenerated electrons; Finally, the active hydrogen species migrate to the surface of the active nitrobenzene molecule to form aniline. The improved light harvesting efficiency, suppressed electron-hole recombination rate together with the more available active sites, accounted for the better catalytic performance of CoS_2_/graphene in photocatalytic SHN.

### 2.3 Ce_2_S_3_

Ce_2_S_3_ is a semiconductor with BG value of 2.1 eV, CB of −0.91 eV and VB of 1.19 eV, respectively (Xu and Schoonen, 2000; Ranjith et al., 2016; Xu et al., 2021; Jia et al., 2022; Rauf et al., 2022). The highly negative potential of CB allows Ce_2_S_3_ as an ideal photocatalyst for SHN (Kudo and Miseki, 2008). Chen et al. (2013) synthesized a Ce_2_S_3_ catalyst by the coprecipitation method, which realized a nitrobenzene conversion and a selectivity towards aniline of 36.2% and 43.9%, respectively, under the conditions of 0.56 g (4.0 g L^−1^) catalyst, 30°C_,_ 375 W mercury lamp irradiation and 5.0 h reaction time.

The evaluation conditions influence greatly on the performance of Ce_2_S_3_ in SHN. ➀ Catalyst concentration. When the catalyst concentration was lower than 4.0 g L^−1^, the catalytic performance increased with the increasing of the photocatalyst dosage, owing to the improved utilization of photons; However, light scattering and shielding effects occurred when the catalyst concentration exceeded 4.0 g L^−1^, which resulted in the ineffective utilization of Ce_2_S_3_ and photons. Therefore, the optimal catalyst concentration was determined as 4.0 g L^−1^. ➁ Hole scavenger. Methanol is a better hole scavenger than others (such as ethanol and isopropanol) because of its advantages, such as lower viscosity, higher polarity, lower polarizability as well as easier to be captured by photoexcited holes.

**FIGURE 7:**
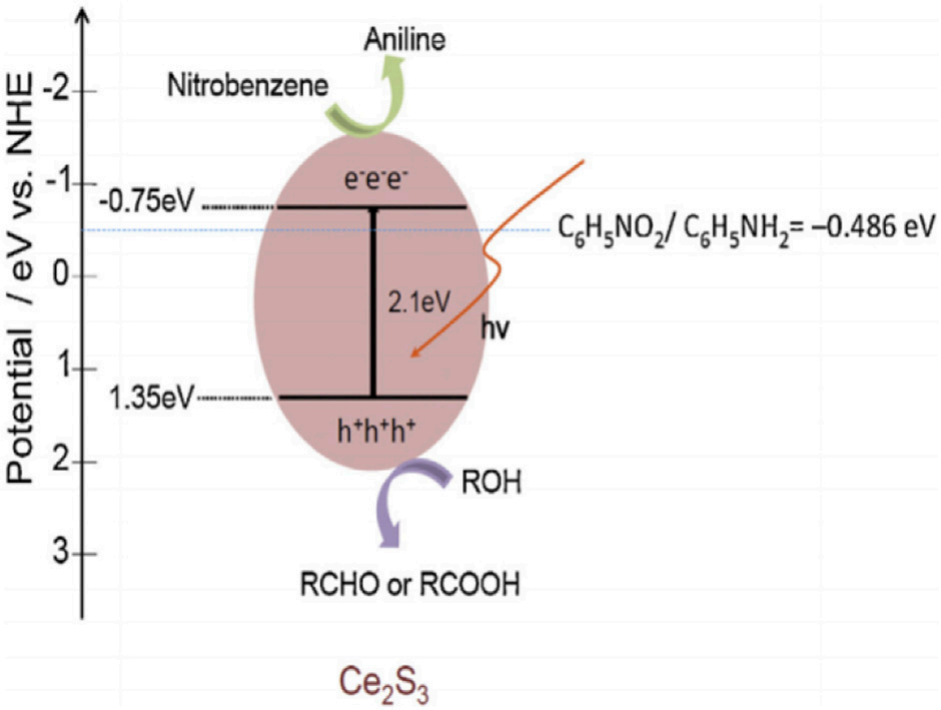
Mechanism of SHN catalyzed by Ce_2_S_3_ photocatalyst. Reproduced with permission from reference (Chen et al., 2013).

According to the energy band structure of Ce_2_S_3_ calculated by empirical equation (Eqs 1, 2), the approximate reaction mechanism is inferred (Figure 7).

$$E_{VB}=X-E_{e}+0.5E_{g}$$ (1)

$$E_{CB}=E_{VB}-E_{g}$$ (2)

(where E_VB_ is the VB edge potential, X is the electronegativity of the semiconductor, E_e_ is the standard hydrogen electrode potential, and E_g_ is the BG energy of the semiconductor.)

The photocatalytic reduction of nitrobenzene occurs on the surface of Ce_2_S_3_, and photogenerated electron hole pairs will be generated under illumination. In order to prevent the recombination of electron hole pairs, methanol is adsorbed on the surface of Ce_2_S_3_ to capture holes to reduce the recombination rate, increase the probability of photoexcited electrons adsorbed on Ce_2_S_3_, and improve the conversion rate of nitrobenzene reduction to aniline (Figure 7).

### 2.4 CeO_2_

**FIGURE 8:**
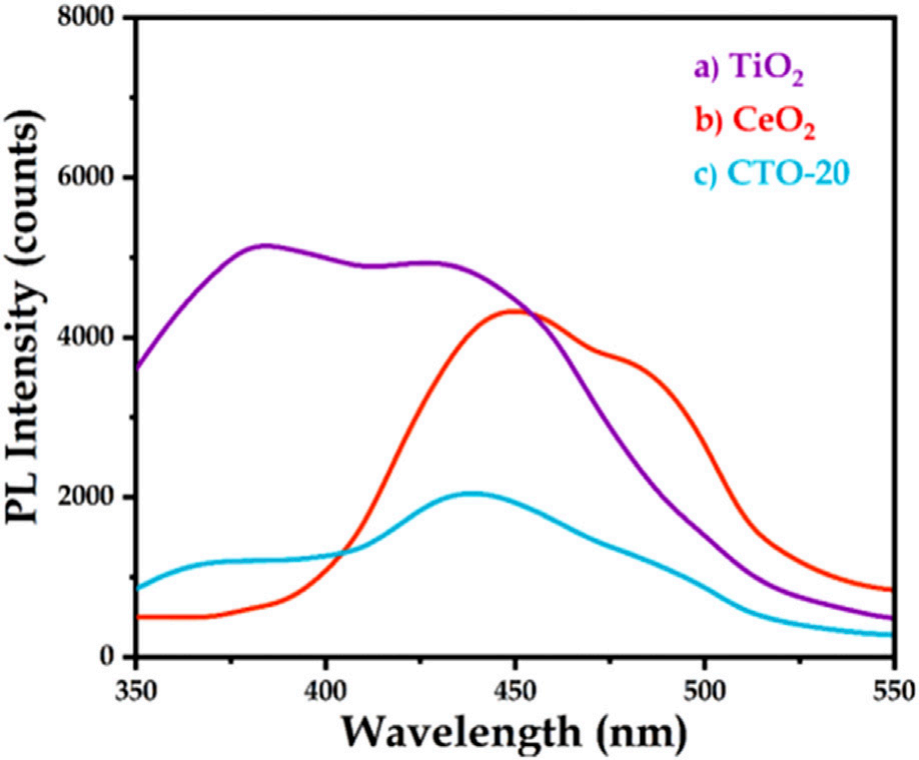
PL spectra of TiO_2_, CTO-20, and CeO_2_ nanomaterials. Reproduced with permission from reference (Lu et al., 2021)

The BG of CeO_2_ is about 3.19 eV. In addition, CeO_2_ has a dynamically reversible Ce^3+^/Ce^4+^ redox site. It is one of the promising catalysts or promoters in hydrogenation of nitrobenzene, since it is beneficial for the adsorption of nitro groups on its surface (Huang et al., 2021). Based on this background, Lu et al. (2021) prepared CeO_2_ modified TiO_2_ nanocomposites by sol-gel method, and evaluated their performance in photocatalytic hydrogenation of nitrobenzene. It revealed that, CeO_2_-TiO_2_ (the weight ratio of CeO_2_ was 20%, short for CTO-20) could realize a nitrobenzene conversion and a selectivity towards aniline of 98.0%, under the conditions of 20.0 mg catalyst, 1.0 mmol nitrobenzene, 2.0 mmol hydrazine hydrate, 4.0 mL methanol, 25°C, 300 W Xenon lamp irradiation and reaction time 4.0 h. Compared with pure TiO_2_ and pure CeO_2_, CTO-20 nanocomposites had a larger amount of oxygen vacancies, inhibited the combination of electron-hole pairs (Figure 8) and exhibited the highest catalytic activity. Characterization revealed that, the photogenerated electrons in the CB fell into the energy level of the oxygen vacancy through the non-irradiation process, and then recombined with the photogenerated holes in the VB, accompanied by fluorescence emission. Thus the recombination of the photogenerated carriers was suppressed. Based on the energy band structure of CeO_2_ and TiO_2_, the possible mechanism of hydrogenation of nitrobenzene under visible light was proposed: photogenerated electrons were excited to CB, leaving holes at VB of TiO_2_, and photogenerated holes at VB of TiO_2_ migrated to VB of CeO_2_. Here, methanol in the reaction system acted as a hole scavenger, trapping the holes on CeO_2_ VB, preventing the recombination of carriers. Therefore, the photogenerated holes of TiO_2_ at VB has strong oxidation ability, which can effectively oxidize and split hydrazine hydrate into protons and electrons. Then nitrobenzene was reduced by electrons and protons at the CB of TiO_2_, and aniline was obtained as the final product under visible light irradiation.

### 2.5 CdS

CdS has a bandgap of 2.4 eV and is strong in harvesting visible light (Jingrun et al., 2014; Yu et al., 2019; Li et al., 2020; Cui et al., 2021). However, as a photocatalyst, CdS suffers from the following disadvantages, ➀ the photogenerated electrons and holes recombine quickly, resulting in low quantum efficiency for solar light utilization, and ➁ the photostability of CdS is relatively poor (Zhang et al., 2011; Eskandari et al., 2014; Gao et al., 2015; Zhang et al., 2019a). To overcome the shortages of CdS, several heterojunction/hybrid catalysts have been designed.

Xiao et al. (2014) and Ye et al. (2017) studies are representatives. Xiao et al. (2014) constructed a graphene nanosheets-CdS quantum dots composite films and adopted it for the selective reduction of various halogenated nitroaromatic hydrocarbons under visible light conditions. Under the condition of 15.0 mL (10.0 mg L^-1^) nitroaromatic, 20.0 mg HCO_2_NH_4_, 25°C and 300 W Xe lamp irradiation, after evaluating for 3.0 h, the conversion rate of various nitroaromatic substances (such as 1-chloro-4-nitrobenzene, 1-bromo-4-nitrobenzene and 4-nitrotoluene etc.) reached about 80.0%. The high activity was mainly due to the capture of photo-excited electrons by graphene nanosheets, which significantly enhanced the adsorption of substrates, facilitated electron hole separation and consequently increased the possibility of photo-induced electrons participating in photocatalytic hydrogenation. Ye et al. (2017) fabricated a CdS/graphitic carbon nitride (CdS/g-C_3_N_4_) heterostructure catalyst for SHN research. It revealed that, in photocatalytic SHN, over CdS/g-C_3_N_4_, under the conditions of 0.1 g catalyst, 15.0 mL benzotrifluoride solution (benzyl alcohol and nitrobenzene), 60°C and 300 W Xe lamp irradiation, after evaluating for 4.0 h, nitrobenzene conversion and the selectivity towards aniline reached 49.2% and 52.8%, respectively, much higher selectivity than those on CdS (18.0% and 37.5%, respectively) and g-C_3_N_4_ (11.0% and 40.0%, respectively). Characterization suggested that the edge of the absorption band of CdS/g-C_3_N_4_ was red-shifted compared with that of pure g-C_3_N_4_, which was beneficial for harnessing solar energy. Meanwhile, the introduction of g-C_3_N_4_ limits the growth of cadmium sulfide particles, resulting in a larger specific size and specific surface area of CdS/g-C_3_N_4_, which has higher catalytic activity. In addition, there was a strong interaction between CdS and g-C_3_N_4_, which was conducive to the efficient separation and migration of photoexcited carriers, thereby improving the photocatalytic activity.

### 2.6 g-C_3_N_4_

**FIGURE 9:**
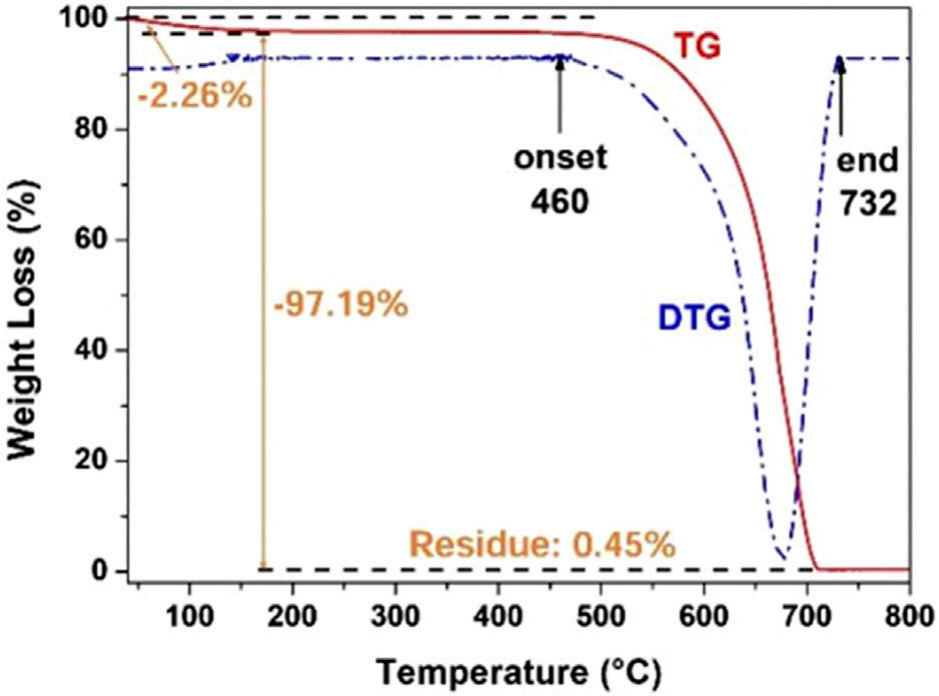
TG/DTG curves of the synthesized g-C_3_N_4_ catalyst in air. Reproduced with permission from reference (Xiao et al., 2018).

As a semiconductor, g-C_3_N_4_ has a unique electronic energy band and visible light activity with a BG of about 2.7 eV. In addition, g-C_3_N_4_ is non-toxic and cheap, with excellent physical and chemical stability (Ismael, 2020; Li et al., 2022a; Wudil et al., 2023). The merits of g-C_3_N_4_ make it as an excellent photocatalyst in visible light region. For example, Xiao et al. (2018) synthesized a polymerized g-C_3_N_4_ photocatalyst *via* thermally condensing urea at high temperatures. The polymerized g-C_3_N_4_ catalyst had a unique structure with a loose and porous sheet-like morphology, and specific surface area of 74.2 m^2^ g^−1^. In addition, thermogravimetric analysis (TGA) revealed an onset temperature of g-C_3_N_4_ pyrolysis as high as 460°C (Figure 9), indicating that the synthesized g-C_3_N_4_ had excellent chemical stability in air at temperatures lower than 460°C. In photocatalytic SHN, over g-C_3_N_4_, under the conditions of 20.0 mg catalyst, 0.2 mmol nitrobenzene, 2.0 mL H_2_O, 1.0 mmol hydrazine hydrate, 90°C and 300 W Xe lamp irradiation, after evaluating for 20.0 h, nitrobenzene conversion and the selectivity towards aniline reached 100.0% and >99.0%, respectively. Mechanism study indicated that, when the transient electron hole pair was generated on the g-C_3_N_4_ surface upon light irradiation, the generated photoelectrons were transferred to the nitrobenzene molecules adsorbed on g-C_3_N_4_, and hydrazine (a strong reducing agent) effectively removed the holes, thus strengthening the segregation of charge carriers and accelerating the hydrogenation of nitrobenzene to aniline.

### 2.7 Polymeric carbon nitride (PCN)

PCN has the advantages of easy synthesis, visible light sensitivity and suitable energy bands. During the past few years, it has been successfully used in the hydrogenation of nitrobenzene (Wang et al., 2018a; Liao et al., 2021; Patel et al., 2021). Generally speaking, pristine PCN exhibits poor catalytic activity and modification is necessary to achieve high catalytic performance.

**FIGURE 10:**
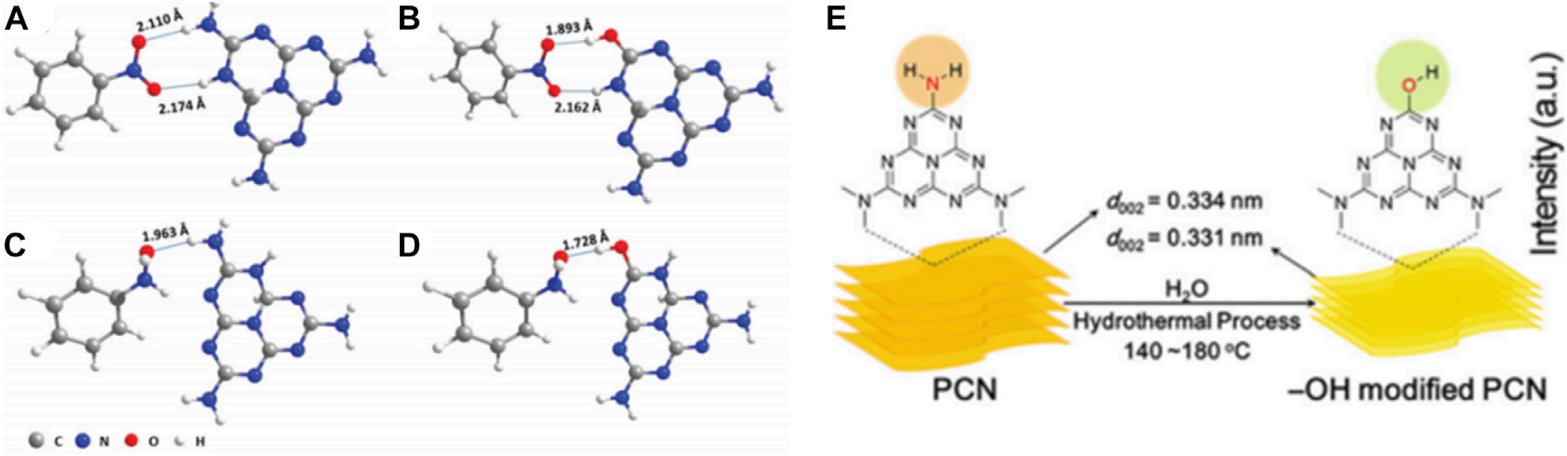
Reaction mechanism model diagram. **(A, C)** The best adsorption states of nitrobenzene and PHA on PCN, **(B, D)** the best adsorption states of PCN modified by -OH for nitrobenzene and PHA (where the numbers indicate the length of hydrogen bond), and **(E)** schematic diagram of hydroxyl modified PCN. Reproduced with permission from reference (Pei et al., 2021).

For instance, Pei et al. (2021) designed a surface -OH group-modified PCN (denoted as PCN-160, since it was treated at 160°C) *via* a simple green hydrothermal treatment method, which could realize a nitrobenzene conversion and a selectivity towards N-phenylhydroxylamine (PHA) of 98.0% and 80.0%, under the conditions of 50.0 mg catalyst, 30.0 μmol nitrobenzene, 1.5 mL isopropanol, 60°C, 100 W white light emitting diode (LED) lamp irradiation and reaction time 18.0 h. DFT calculations showed that the interaction energies of PHA and nitrobenzene were 0.57 eV and 0.40 eV on PCN. Through the hydrogen bonding interaction, PCN-160 energy was enhanced by 0.12 eV and 0.42 eV, respectively. In PCN-160, the O—H···O hydrogen bond replaced the O—H···N hydrogen bond, where the bond length of O—H···O was shorter than O—H···N. The significant change in energy and a decrease in hydrogen bond length (from 2.110 to 1.893 Å, 1.963 to 1.782 Å) between nitrobenzene and the active center -OH favored the hydrogenation of nitrobenzene (Figures 10A–D). Over PCN-160, ① the introduction of -OH functional groups shifted the CB of PCN to a suitable position, thus reducing the recombination probability during electron transfer and providing high-energy photogenerated electrons to promote the reduction of nitrobenzene; and ② the introduced -OH groups facilitated the adsorption of nitrobenzene, reduced the recombination velocity of electron hole pairs, and provided a large number of protons to participate in the hydrogen transfer process. The above two factors worked synergistically to achieve the cumulation of PHA and ensure high selectivity of the reaction (Figure 10E).

### 2.8 Metal-organic frameworks (MOFs)

MOFs are a kind of coordination polymers, which have burgeoned in recent decades and are extensively served as catalysts. MOFs have a three-dimensional pore structure, in which metal ions are generally used as attachment points to support organic ligands to form spatial 3D extensions (Wang et al., 2017; Amarajothi et al., 2018; Zhang et al., 2019b; Li et al., 2022b). The MOFs’ crystal dimensions, conformation, and crystal texture can significantly tailor the visible light absorption capacity, charge separation efficiency, and directional electric charge transport (Kouser et al., 2023).

**FIGURE 11:**
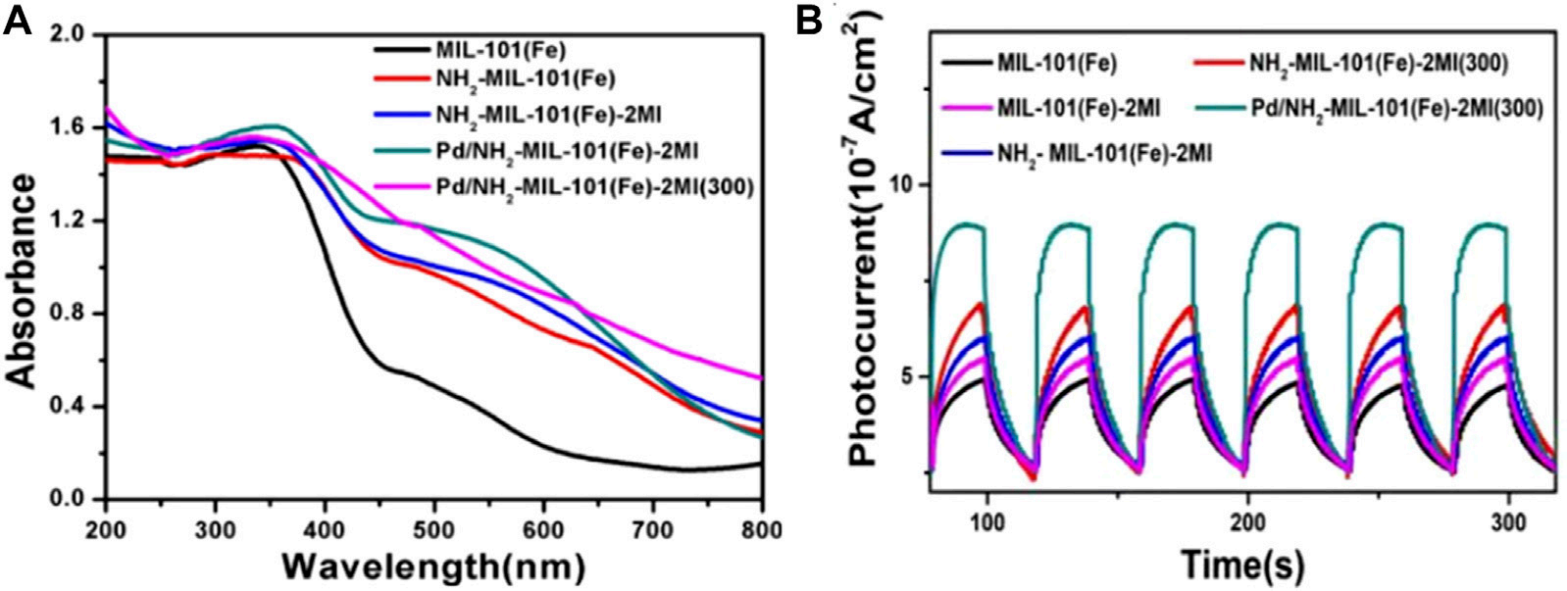
MOF and Pd/MOF catalysts. **(A)** UV-Vis diffuse reflectance spectra, and **(B)** photocurrent response. Reproduced with permission from reference (Cheng et al., 2020).

Cheng et al. (2020) study is representative for the application of MOFs in photocatalytic SHN. They embedded Pd nanoparticles in the cavities of assorted Fe-MOFs (MIL-101(Fe)-2MI, NH_2_-MIL-101(Fe)-2MI and etc.) *via* impregnation method and calcined them in nitrogen to form “quasi-MOF” catalysts (Pd/MIL-101(Fe)-2MI(300), Pd/NH_2_-MIL-101(Fe)-2MI(300), etc., (where “300” represents the calcination temperature). Over Pd/NH_2_-MIL-101(Fe)-2MI(300), under the conditions of 0.1 mmol% catalyst, 0.1 mmol nitrobenzene, 3.0 mmol benzyl alcohol, 2.0 mL CH_3_CN and 0.75 W cm^−2^ blue LED irradiation, after evaluating for 24.0 h, nitrobenzene conversion and the selectivity towards N-benzyl aniline reached 100.0% and 85.0%, respectively (Cheng et al., 2020). UV-vis diffuse reflectance spectra (Figure 11A) suggested that all the catalysts were capable for harvesting visible light, which proved their feasibility as photocatalysts. In addition, Pd/NH_2_-MIL-101(Fe)-2MI(300) with amino groups, 2MI and the loading of Pd nanoparticles, exhibited the highest photocurrent (Figure 11B), indicating supreme charge transfer efficiency and minimum charge transfer electric resistance. The light harvesting capacity as well as the highest photocurrent responded for the high catalytic performance of Pd/NH_2_-MIL-101(Fe)-2MI(300).

## 3 Plasmonic-metal based catalysts

Plasmonic-metal based photocatalysts with localized surface plasmon resonance (LSPR) effects are gradually emerging because of their capacity to harness an ultra-wide spectrum of sunlight. LSPR is a physical optical phenomenon. It uses the evanescent wave that penetrates the metallic membrane when the light is fully reflected at the interface between the glass and the metal film, which induces free electrons in the metal to generate surface plasmons (Xie et al., 2022). LSPR effect mainly exists in plasmonic metals, such as Au, Cu and Ag (Zheng et al., 2014; Kavitha and Kumar, 2019; Wang et al., 2021; Silva et al., 2022). Efficiently identifying and directing energy pathways from the plasmonic metals to substrates is considered a key to achieve improved catalytic efficiency (Gellé and Moores, 2019; Silva et al., 2022). In this subsection, we will describe the applications of plasmonic-metal based catalysts in photocatalytic SHN.

### 3.1 Au-based catalysts

**FIGURE 12:**
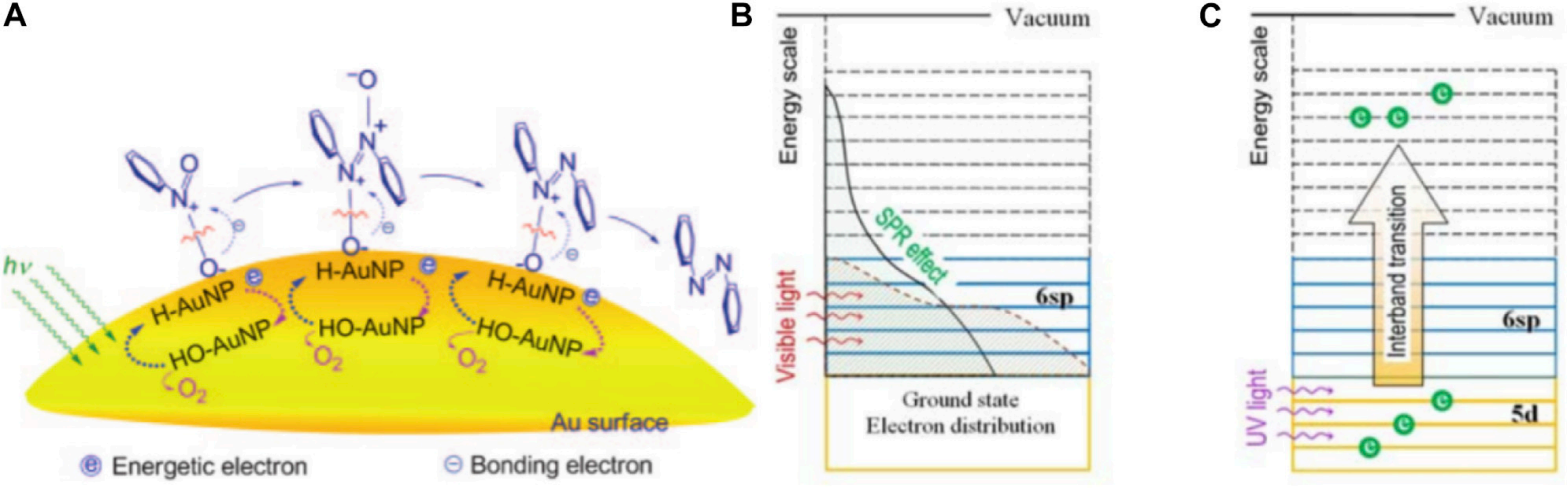
**(A)** Mechanism for the photocatalytic SHN over Au/ZrO_2_, **(B)** electron excitation in Au nanoparticles due to the LSPR effect when illuminated with visible light, and **(C)** an interband transition when illuminated with UV light. Reproduced with permission from reference (Zhu et al., 2010).

Huang et al. (2021) investigated the feasibility of Au-based catalysts in photocatalytic SHN. They prepared a series of Au/ZrO_2_ catalysts with different Au loadings (1.5, 3.0, and 5.0 wt%) by supporting Au nanoparticles on ZrO_2_ powder (Zhu et al., 2010). It revealed that, in photocatalytic SHN, under the conditions of 100.0 mg catalyst, 3.0 mmol nitrobenzene, 30.0 mL isopropyl alcohol, 0.3 mmol KOH and 40°C, after evaluating for 5.0 h, 3.0% Au/ZrO_2_ gave a nitrobenzene conversion of 100.0% and a selectivity towards azobenzene of >99.0%, respectively, much more active than 1.5% Au/ZrO_2_ (59.0% and 88.0%, respectively) and 5.0% Au/ZrO_2_ (69.0% and 82.0%, respectively). 1.5% Au/ZrO_2_ was inferior owing to the less available active sites. On the other hand, over 5.0% Au/ZrO_2_, the excessive Au loading led to the aggregation of Au nanoparticles and reduced the specific surface area of Au nanoparticles, where the catalytic reaction took place. In photocatalytic SHN, H-Au species were formed on the surface of Au nanoparticles, which were able to combine with the oxygen atoms of the N—O bond to generate HO-Au and achieve electrophilic N—O bond cleavage. Au nanoparticles absorbed visible light through the LSPR effect, resulting in changes of the electron distribution across energy levels and providing energy to cleave the N—O bond (Figure 12A). E.g., Au 6 sp electrons could gain energy through LSPR effect and migrate to higher energy levels (Figure 12B) and high-energy electrons could also be generated through electron interband excitation from Au 5d to Au 6 sp under UV light irradiation (Figure 12C).

### 3.2 Ag-based catalysts

**FIGURE 13:**
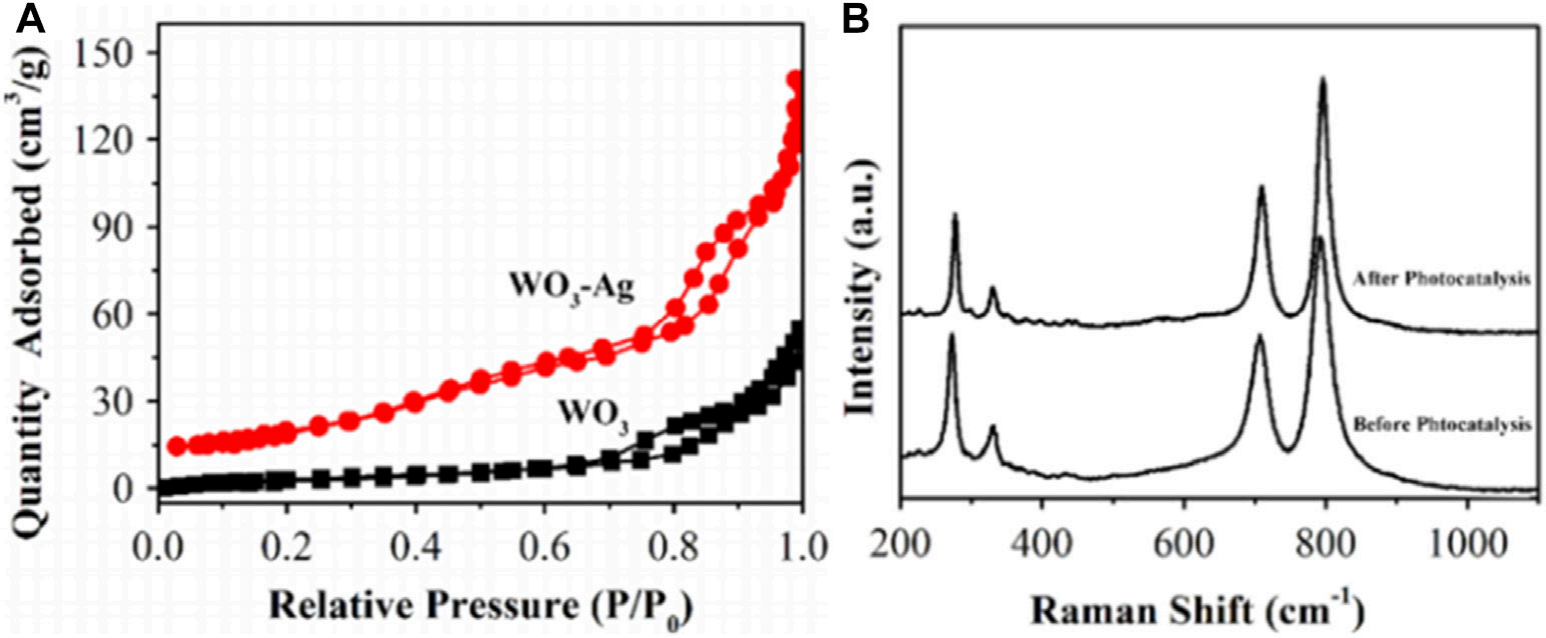
**(A)** N_2_ adsorption-desorption isotherms of WO_3_ nanowires and WO_3_-Ag, and **(B)** Raman spectrum of WO_3_-Ag before and after stability test. Reproduced with permission from reference (Li et al., 2015).

Plasmonic properties of Ag endow Ag-based catalysts unique catalytic properties with high performance in various catalytic reactions (Wang et al., 2018b; Chaudhary and Ingole, 2018; Ojha et al., 2021; Zhao et al., 2021; Dutta et al., 2022; Shi et al., 2022). Ag-based catalysts have also been successfully applied in photocatalytic SHN. For instance, by taking advantages of the plasmonic properties of Ag as well as the high thermal and physicochemical stability of n-type semiconductor WO_3_, Li et al. prepared WO_3_-Ag nanowires by facial chemical method, where WO_3_-Ag was formed by connecting massive WO_3_ nanowires and slende Ag nanowires, with a multi-aperture network structure (Li et al., 2015). In photocatalytic SHN, under the conditions of 50.0 mg catalyst, 50.0 mL nitrobenzene-methanol solution, after evaluating for 2.0 h, WO_3_-Ag gave a nitrobenzene conversion of 94.0%, which was much better than other catalysts (Ag and WO_3_). WO_3_ nanowires and WO_3_-Ag nanowires exhibited similar isotherms with H_3_-type hysteresis loops (Figure 13A). By comparison, WO_3_-Ag exhibited a large specific surface area (77.1 m^2^ g^−1^) while the specific surface area of WO_3_ was 29.9 m^2^ g^−1^. It indicated that the addition of Ag greatly increased the specific surface area of WO_3_, which offered more active sites and promoted the photocatalytic reaction. In WO_3_-Ag photocatalyst, Ag is a plasmonic metal. Ag can expand the light absorption to a longer wavelength and excite the electron-hole pairs in WO_3_ by transferring the plasmon energy. Moreover, the energy of electrons or holes is transferred from plasmonic metal Ag to semiconductor with energy lower than the BG. The physical and chemical properties of the catalyst change, thus improving the activity and stability of the catalyst. Moreover, WO_3_-Ag nanowires still remained more than 90.0% of the initial activity after four cycles. The spectra before and after cycling suggested that the catalyst structure remained basically unchanged, further confirming the structural stability of WO_3_-Ag (Figure 13B).

### 3.3 Cu-based catalysts

Au- and Ag- based catalysts are the most extensively studied noble metal catalysts in photocatalytic reactions. Unfortunately, the high cost of Au and Ag seriously hindered the realization of industrial production. On the contrary, non-precious Cu-based catalysts exhibit excellent LSPR effects and have great potential to replace noble metals in photocatalytic reactions (Araújo et al., 2019; Xin et al., 2021).

**FIGURE 14:**
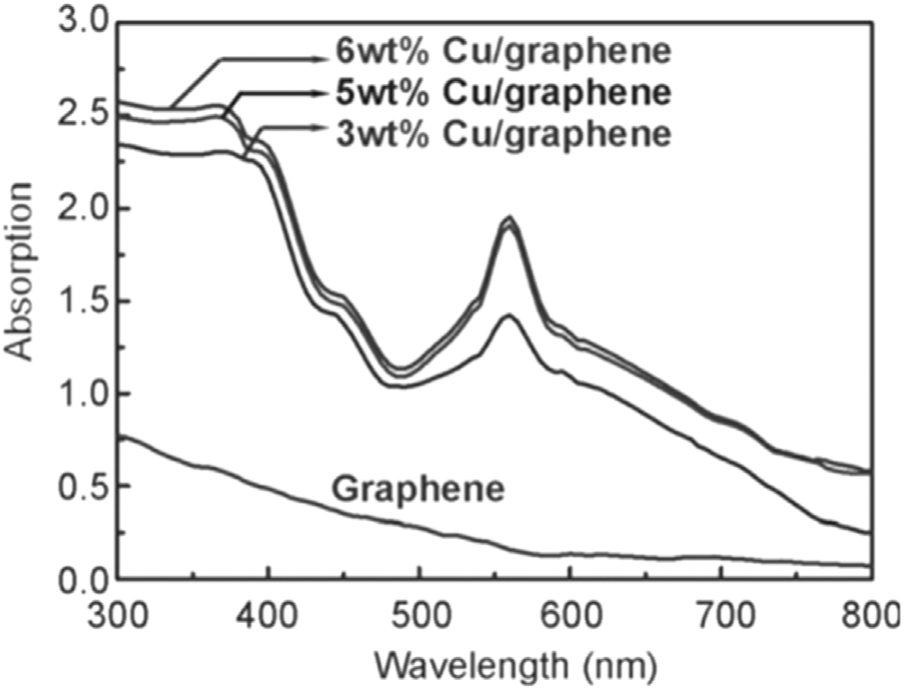
UV-vis absorption spectra of Cu/graphene photocatalysts. Reproduced with permission from reference (Guo et al., 2014).

Cu nanoparticles are of poor chemical stability, because they readily oxidize to CuO or Cu_2_O in air or in the presence of trace amounts O_2_. Guo et al. (2014) found that the electronic structure of Cu atoms on graphene could be affected by carbon vacancies and dangling bonds in graphene, and ameliorated its chemical stability. Based on this discovery, they prepared a series of Cu/graphene catalysts with different Cu loadings (3.0, 5.0, and 6.0 wt%) for SHN. It revealed that, in photocatalytic SHN, under the conditions of 100.0 mg catalyst, 3.0 mmol nitrobenzene, 30.0 mL isopropyl alcohol, 0.3 mmol KOH, 300 W Xe lamp irradiation and 90°C, after evaluating for 5.0 h, 5.0% Cu/graphene gave a nitrobenzene conversion of 98.0% and a selectivity towards azobenzene of 98.0%, respectively, much more active than 3.0% Cu/graphene and 6.0% Cu/graphene. 3.0% Cu/graphene exhibited significantly weaker LSPR absorption due to the low content of Cu (Figure 14). The Cu particles in 6.0% Cu/graphene (40.3 nm) were much larger than those in 5.0% Cu/graphene (15.4 nm), which resulted in smaller specific surface area and fewer active centers than 5.0% Cu/graphene. In photocatalytic SHN, isopropanol acted as a hydrogen source and reaction solvent, and KOH enhanced the hydrogen release of isopropanol. The released hydrogen bonds formed H-Cu species on Cu surfaces, which produced HO-Cu by trapping oxygen atoms in N-O bonds, resulting in azobenzene. The high-energy electrons excited by the Cu LSPR effect strongly interacted with the electrophilic nitro group in the nitrobenzene, which promoted the cleavage of the N-O bond and accelerated the reaction.

**FIGURE 15:**
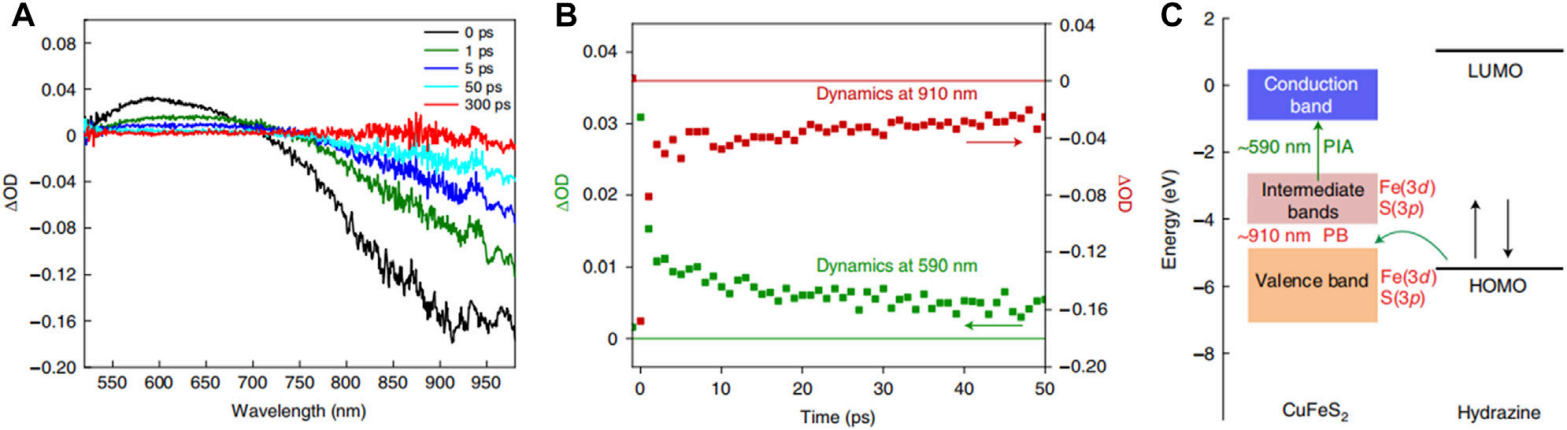
**(A)** Time-resolved TAS of CuFeS_2_ catalysts, **(B)** the transient dynamics of CuFeS_2_ PIA at 590 nm and PB at 910 nm, and **(C)** diagrammatic sketch of energy level diagrams of CuFeS_2_ and hydrazine. Reproduced with permission from reference (Poulose et al., 2022).

In addition to metallic Cu, chalcopyrite (CuFeS_2_), which is a natural mineral, exhibits LSPR properties (Bhattacharyya and Pandey, 2016; Ghosh et al., 2016; Sugathan et al., 2018). Poulose et al. (2022) adopted sunlight as the only energy input, CuFeS_2_ as the catalyst, and hydrazine as the solvent to provide protons and electrons for photocatalytic SHN. Over CuFeS_2_, under the conditions of 10.0 mg catalyst, 0.1 mmol nitrobenzene, 0.8 mL hydrazine, 3.0 mmol ethanol and room temperature, after evaluating for 2.0 h, both the conversion of nitrobenzene and the selectivity to aniline reached 100.0%. When the reaction was carried out at 40°C without light irradiation, the yield of aniline reached 44.1%, indicating the intrinsic activity of CuFeS_2_ was mainly stemmed from the photocatalytic process. Transient absorption spectroscopy (TAS) study revealed two main processes (Wang et al., 2015), photoinduced absorption (PIA) and (Maji et al., 2011) photobleaching (PB) features, respectively (Figures 15A, B). The PIA curve and PB feature observed at the same time was attributed to the transition from the temporary occupation state in the median to CB, and the transition from depleted VB to the state in the median, respectively. Among them, the excess energy of carrier cooling excited electrons was transferred to the lattice, which eventually led to the heating of nanocrystals. It indicated the formation of holes in the VB of CuFeS_2_ and hot electrons was formed in the intermediate band and CB of CuFeS_2_ (Figure 15C). This energy matching promoted the good interaction between the highest occupied molecular orbital of hydrazine and the VB holes of CuFeS_2_, which was considered as the main reason for the excellent performance of CuFeS_2_. In addition, there was synergy between the metal centers, Fe and Cu in CuFeS_2_. Fe central site was used to bind and activate hydrazine to form instantaneous spin active species, [H(FeS_2_)NH-NH_2_], which delivered protons and electrons to the adjacent Cu(I)S_2_ site. Cu(I)S_2_ sites interacted with the nitro-substrate to produce the N-phenylhydroxylamine radical. For these reasons, CuFeS_2_ can not only form optical excitation complexes with the reactants but also possess a particularly high reaction rate.

## 4 Dye as photocatalyst

Organic dyes produce excited states by absorbing visible light and making transitions (dye*). Then electrons transfer to dye* to form dye^+**·**^ radical and reactant radical anion. After that, reactant radial anion is reduced to the target product and dye is regenerated from dye^+**·**^ radical by scavenger. A key feature of dye as photocatalyst is that their energy levels (i.e., highest occupied molecular orbital) are more flexible and tunable (Zhang and Zhu, 2005; Lang et al., 2014; Franchi and Amara, 2020; Yang et al., 2022). Therefore, dye-catalyzed systems are promising for photocatalytic SHN.

**FIGURE 16:**
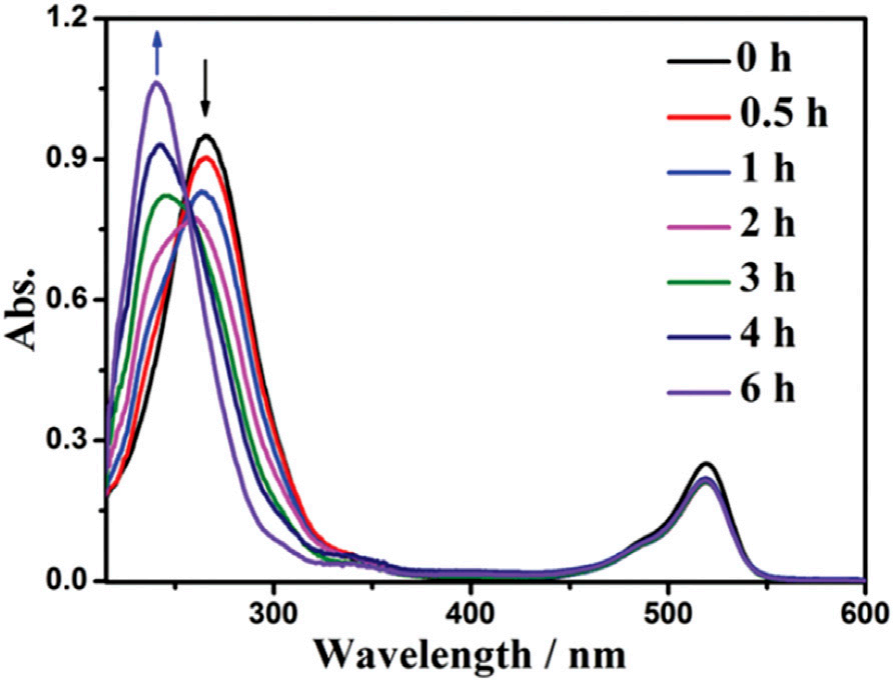
Changes of UV-vis absorption spectrum of nitrobenzene solution during irradiation. Reproduced with permission from (Yang et al., 2014).

Eosin Y (EY) is a chemically synthesized acid dye and has been widely utilized as catalyst due to its simple structure, low price, and good effect (Liu et al., 2020; Dhara et al., 2021). For instance, Yang et al. (2014) reported a simple and efficient catalytic hydrogenation reduction reaction of nitrobenzene with EY as catalyst and triethanolamine (TEOA) as reducing agent under the illumination of green LED light. Under the conditions of EY (1.0 mol%) catalyst, 0.2 mmol nitrobenzene, EtOH/H_2_O (3:2) of 5.0 mL, pH 8.5 and room temperature, after evaluating for 8.0 h, nitrobenzene conversion and the selectivity towards aniline reached 100.0% and 93.0%, respectively. The absorption peak of λ_max_ at 270 nm is the absorption of light by nitrobenzene, which gradually decreases with time. The absorption peak of λ_max_ at 240 nm is the absorption of light by aniline. The transfer of the peak indicates that nitrobenzene is successfully converted into aniline. In the photocatalytic reaction, the position of the absorption peak and the intensity of λ_max_ at 520 nm remained almost unchanged, which indicated that EY was very stable (Figure 16). It may be due to the formation of EY cationic radicals (EY^+**·**^), which makes the electron transfer from TEOA to EY^+**·**^, and leads to the rapid regeneration of EY. After a series of control experiments, it was found that there were two important factors affecting the photocatalytic performance of EY in SHN. ① The pH range of the catalytic system. In this photocatalytic system, the optimal pH range was about 8.5, TEOA acted as a sacrificial electron donor, providing electrons and protons for reduction reactions. In strong alkaline solutions, the reduction efficiency was very low since a limited number of protons was available for the reduction process. On the contrary, in strong acid solution, TEOA would be protonated, thereby reducing its electron donation ability and efficiency. ② The dosage of TEOA. In the reduction process, each TEOA molecule can contribute two electrons and two protons. Therefore, when three equivalent TEOA was used, full conversion can be achieved. The mechanism on photocatalytic SHN by EY was proposed. For the photoinduced electron transfer from the triplet EY to nitrobenzene in the charge transfer route, EY^+**·**^ and nitrobenzene radical anions will be formed. At the same time, the electron transfer from TEOA to EY^+**·**^ will regenerate EY and give TEOA^+**·**^, and then the nitrobenzene radical anion reacts with TEOA to finally produce aniline.

## 5 Summary and outlooks

In the past few years, researchers have carried out extensive research in order to find efficient photocatalysts for SHN. Semiconductor, plasmonic metal-based catalyst and dye have been reported active in SHN. This paper summarizes the research progress of various catalysts for SHN, with special attention on semiconductor-based catalysts. Table 1 lists the performance of some typical catalysts.

**TABLE 1:** The performance of some typical catalysts in SHN reaction.

Catalysts	Catalyst type	Reaction conditions	Product yield (%)	Ref.
Rutile TiO_2_	Semiconductor	Catalyst: 5.0 mg, 2-PrOH: 5.0 mL, nitrobenzene: 10.0 mmol, illumination: 500 W, *λ* > 300 nm, Xe lamp.	97.0	Shiraishi et al. (2012)
TiO_2_@N-AC	Semiconductor	Catalyst: 10.0 mg, nitrobenzene: 0.08 mmol, isopropanol: 4 mL, temperature: 30°C, irradiation: 300W, *λ* > 350 nm, Xe lamp, reaction time: 6.0 h	98.8	Xu et al. (2019)
Pt-TiO_2_-RGO	Semiconductor	Catalyst: 50.0 mg, nitrobenzene: 50.0 mL, triethanolamine: 0.6 mL, room temperature, illumination: 300 W Xe lamp irradiation, reaction time: 20.0 h	99.0	Qiu et al. (2016)
CoS_2_/graphene	Semiconductor	Catalyst: 40.0 mg, nitrobenzene: 1.0 mmol, ethanol: 10.0 mL, temperature: 30°C, 0.25 MPa H_2_, illumination: 300 W Xe lamp, reaction time: 1.5 h	100.0	Ma et al. (2017)
Ce_2_S_3_	Semiconductor	Catalyst: 0.56 g (4.0 g L^-1^), 140 mL 8.13 × 10^−4^ mol L^-1^ of nitrobenzene in methanol solvent, illumination: 375 W Xe lamp, reaction time: 5.0 h	43.9	Chen et al. (2013)
CdS/g-C_3_N_4_	Semiconductor	Catalyst: 0.1 g, benzotrifluoride solution (benzyl alcohol and nitrobenzene): 15.0 mL, temperature: 60°C, illumination: 300 W Xe lamp, reaction time: 4.0 h	52.8	Ye et al. (2017)
g-C_3_N_4_	Semiconductor	Catalyst: 20.0 mg, nitrobenzene: 0.2 mmol, H_2_O: 2.0 mL, hydrazine hydrate: 1.0 mmol, temperature: 90°C, illumination: 300 W Xe lamp, reaction time: 20.0 h	99.0	Xiao et al. (2018)
PCN	Semiconductor	Catalyst: 50.0 mg, nitrobenzene: 30.0 μmol, N-Phenylhydroxylamine: 1.5 mL, illumination: 100 W white LED lamp (0.6 Wcm^−2^), temperature: 60°C, reaction time: 18.0 h	80.0	Pei et al. (2021)
Pd/NH_2_-MIL-101(Fe)-2MI(300)	Semiconductor	Catalyst: 0.1 mmol% (based on Pd), nitrobenzene: 0.1 mmol, benzyl alcohol: 3.0 mmol, CH_3_CN: 2.0 mL, illumination: 0.75 W cm^-2^ blue LED, reaction time: 24.0 h	85.0	Cheng et al. (2020)
Au/ZrO_2_	Plasmonic-metal based catalyst	Catalyst: 100.0 mg, nitrobenzene: 3.0 mmol, isopropyl alcohol: 30.0 mL, KOH: 0.3 mmol, temperature: 40°C, reaction time: 5.0 h	99.0	Zhu et al. (2010)
Cu/graphene	Plasmonic-metal based catalyst	Catalyst: 100.0 mg, nitrobenzene: 3.0 mmol, isopropyl alcohol: 30.0 mL, KOH: 0.3 mmol, illumination: 300 W Xe lamp, temperature: 90°C, reaction time: 5.0 h	98.0	Guo et al. (2014)
CuFeS_2_	Plasmonic-metal based catalyst	Catalyst: 10.0 mg, nitrobenzene: 0.1 mmol, hydrazine: 0.8 mL, ethanol: 3.0 mmol, room temperature, reaction time: 2.0 h	100.0	Poulose et al. (2022)
EY	Dye as catalyst	Catalyst: EY (1.0 mol%), nitrobenzene: 0.2 mmol, EtOH/H_2_O (3:2): 5.0 mL, pH 8.5, room temperature, reaction time: 8.0 h	93.0	Yang et al. (2014)

In conclusion, the catalyst development is still in the initial stage, which is both an opportunity and a challenge, and huge difficulties need to be overcome.

(1) Selective hydrogenation of nitro group is challenging when other functional groups are present in the reactant nitroaromatics. Generally speaking, the hydrogenation reaction preferentially occurs on the thermodynamically more favorable functional group, or multiple unsaturated groups are hydrogenated at the same time, and the degree of hydrogenation cannot be precisely and finely controlled, making it difficult to control specific intermediates and by-products such as hydroxyaniline or azobenzene compounds are occasionally generated. Selective synthesis of hydrogenated products is very difficult, especially to selectively control the reaction pathways to aniline at present. Optimal reaction conditions could inhibit the occurrence of side reactions. For example, increasing the hydrogen pressure could inhibit the desorption of hydroxyaniline and lead it further hydrogenate to aniline, which could avoid the formation of hydroxyaniline as a by-product. Similarly, low reaction temperature can suppress the formation of azobenzene.
(2) An increase in activity is often accompanied by a decrease in selectivity, thus, it is challenging to realize high activity and high selectivity simultaneously. In this regard, there are still difficulties that have not been overcome for most catalysts. Compared with selectivity, conversion can be corrected by increasing process parameters (such as time and temperature), and selectivity promotion is more difficult and important. Future research needs to focus on in-depth study of the structure and distribution of catalyst active centers, increase the contact area between reactants and catalysts, reduce the occurrence of side reactions, and control the concentration gradient of reactants and the reaction at the kinetic level. Reasonable reaction conditions should be explored and suitable catalysts should be designed.
(3) Although noble metals, such as Au, Pt, and Pd, exhibit good catalytic activity, they cannot be applied in a large scale in industry due to their high cost. Therefore, the search for low-cost and high-performance catalysts has become an inevitable trend. In the follow-up research, under the premise of maintaining good conversion effect, it is necessary to focus on the development of new catalysts with simple preparation, low price, good stability and high activity.
(4) Most catalysts exhibit good performance under UV light irradiation, but they are not ideal under visible light irradiation. Therefore, enhancing the light harvesting capacities and widening the light absorption spectrum of the catalysts will be the focus of future research on photocatalytic SHN reaction. Transition metal complex catalysts and organic highly conjugated catalysts could be excited by visible light and exhibit high efficiency for light absorption, which might be the focus of catalyst development for photocatalytic SHN in the future.
(5) The reaction mechanism and corresponding active sites for the adsorption of reactants, dissociation of hydrogen, and subsequent stepwise hydrogenation remain controversial. More research is needed to focus on catalytic process analysis, with advanced and appropriate instrumentation, innovative work, extensive data studies, etc. For example, *in situ* attenuated total reflection infrared spectroscopy could be used to demonstrate the adsorption of substrates in each step of the hydrogenation reactions; with the assistance of theoretical calculations, TAS can be adopted to deduce the reaction mechanism.
(6) As a hydrogen source, alcohols can realize the equal synergistic conversion of nitrobenzene and alcohol under mild conditions. For example, when glycerol is used as the hydrogen source, it is efficiently converted into high value-added product 1,3-dihydroxyacetone during the synthesis of aniline. In addition, polyols, such as 1,3-propanediol, monosaccharide glucose and fructose can also be directly used as hydrogen sources to reduce nitrobenzene. It provides a new idea for the conversion of biomass to high-value-added fine chemicals.
